# Relationship between thyroid nodules and iodine nutritional status, thyroglobulin (Tg), and other influencing factors: a 1:1 matched case-control study

**DOI:** 10.3389/fendo.2025.1685719

**Published:** 2025-11-25

**Authors:** Qingying Lin, Lijin Wang, Diqun Chen, Jiani Wu, Ying Lan, Qingdong Jin, Yanqing Chen, Zhihui Chen, Muhua Wang

**Affiliations:** 1 The First Hospital of PuTian City, Fujian, China; 2 Department of Endemic Diseases, Fujian Center for Disease Control and Prevention, Fujian, China

**Keywords:** thyroid nodules, iodine nutrition, thyroid function, thyroglobulin, TG

## Abstract

**Background:**

An increasing trend in the prevalence of thyroid nodules has highlighted whether thyroid nodules are related to the level of iodine nutrition.

**Methods:**

This 1:1 matched case-control study was conducted to investigate whether thyroid nodules are related to the level of iodine nutrition. The residents who had lived there for more than 5 years with thyroid nodules composed the case group. All the subjects completed questionnaires, and blood and urine samples were collected.

**Results:**

The MUI of the case group in areas with more than adequate iodine intake was lower than that of the control group (*P*<0.05); only the level of Tg in the case group was higher than that in the control group in areas with adequate iodine intake (*P*<0.05). The conditional logistic regression model revealed that there were significant correlations between a history of thyroid disorders, Tg, TgAb, and thyroid nodules.

**Conclusions:**

In areas with more than adequate iodine intake, a slight increase in urinary iodine may be associated with a lower risk of thyroid nodules. A history of thyroid disorders and increased Tg and TgAb levels are risk factors for thyroid nodules. Tg level detection has certain diagnostic value in distinguishing thyroid nodules.

## Introduction

Iodine is a trace element necessary for the synthesis of thyroxine (T4) and triiodothyronine (T3), which are essential for cell metabolism, growth, and physical development; thus, adequate iodine is critical at all stages of life (1). Iodine deficiency can lead to iodine deficiency disorders (IDDs), including miscarriage, stillbirth, endemic disease, neonatal congenital iodine deficiency syndrome, physical growth retardation in children and adolescents, and endemic goiter (2, 3). In addition, excessive iodine intake is associated with hyperthyroidism or hypothyroidism in some susceptible individuals (4).

To eliminate the harm of iodine deficiency, international organizations, including the World Health Organization and the United Nations Children's Fund (UNICEF), advocated the universal salt iodization (USI) strategy in 1993, which received positive responses from member states and achieved remarkable results. The number of iodine-deficient countries decreased from 110 to 32 in 2012 (1). Since the USI policy was implemented in China in 1995, the harm caused by iodine deficiency has been effectively controlled. Ten years later, the incidence of goiter decreased to less than 5% (5). By 2000, China had basically achieved the goal of eliminating iodine deficiency disorders (2). At present, China is in a state of sustained elimination of iodine deficiency disorders, and the overall iodine nutritional status of the population is at an appropriate level. The latest iodine nutrition survey results in Fujian Province in 2014 revealed that the overall iodine nutritional status of adults and children was at an appropriate level (6).

Thyroid nodules are a common clinical thyroid disease. Most were benign nodules without clinical symptoms; when complicated with abnormal thyroid function, corresponding clinical symptoms might appear. Some patients experience compressive symptoms such as hoarseness, dysphagia, or dyspnea because the nodule compresses surrounding tissues. According to early reports, the prevalence rate of thyroid nodules was 4–7%, of which 5–15% were malignant nodules or hidden thyroid cancer in benign nodules (7, 8). With the development of society in recent years, the detection rate of thyroid nodules has shown an increasing trend (9, 10), reaching 19–67% in the general population. The latest literature reports that the average detection rates of thyroid nodules in China are 38%, 45.2% for women, and 31.2% for men (11). During the same period, the national universal salt iodization strategy was implemented. The sharp increase in the prevalence of thyroid nodules has raised wide concerns about the policy of iodizing salt, which has a certain degree of impact on the sustainable prevention and control of iodine deficiency diseases province-wide and even nationwide. Therefore, it is important and urgent to explore the relationships among thyroid nodules, iodine nutrition levels, and other influencing factors.

Approximately 80% of the iodine in the human body is excreted through urine. The urinary iodine concentration is an indicator that is currently recognized internationally, is low-cost and easy to obtain, and can reflect the recent nutritional status of iodine (12). At present, relevant studies on the relationships among iodized salt, iodine nutritional levels, and thyroid nodules have been conducted at home and abroad, but the conclusions obtained have been inconsistent. Some scholars believe that a high iodine content in salt increases the risk of thyroid cancer (13). As mentioned by Kim HJ, the risk of thyroid nodules increases only when iodine intake is low or extremely excessive in areas with sufficient iodine intake (14).

The literature indicates that factors associated with the incidence of thyroid nodules also include sex, age, obesity, radiation, smoking, alcohol consumption, dietary habits, lifestyle, noise, and other factors, which also have a certain impact on the occurrence of thyroid nodules. When research on the relationship between thyroid nodules and iodine nutritional levels is conducted, the impact of the above factors should be fully considered, and the regional differences in iodine nutritional levels in various regions should also be considered. In 2014, a survey on thyroid diseases was conducted in our province, but B-ultrasound was not used for large-scale epidemiological investigations of thyroid nodules in the population. At present, there is a lack of strong evidence on whether the iodine nutritional status of our province has an impact on the prevalence of thyroid nodules.

This study aimed to explore whether thyroid nodules are related to the iodine nutrition level and other related influencing factors through a 1:1 matched case-control study with a strong causal relationship by matching three confounding factors: sex, age, and region. Moreover, differences between thyroid function indicators with and without thyroid nodules were compared to explore whether the detection of thyroglobulin (Tg) levels has certain significance for the diagnosis of thyroid nodules.

## Methods

### Selection of respondents

**Figure 1:**
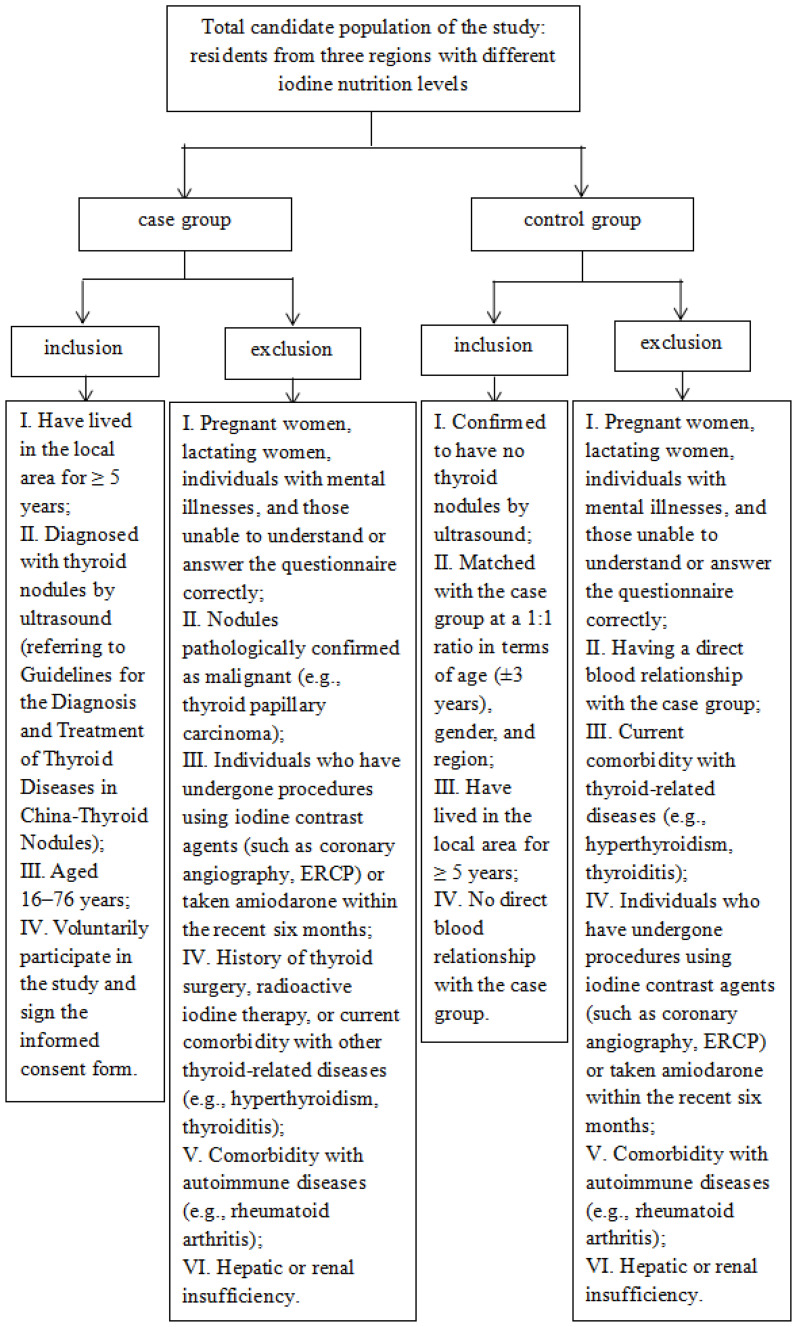
Flow diagram of inclusion and exclusion screening for study subjects.

Villages with median urinary iodine (MUI) concentrations <100 μg/L, 100–199 μg/L, and 200–299 μg/L in the general population were selected as survey sites. The inclusion criteria were as follows: residents whose thyroid nodules were discovered for the first time and who lived in the local area for more than 5 years composed the case group, while the residents who were matched 1:1 with the case group by age (± 3 years), sex, region, and B-ultrasound examination with no nodules composed the control group. The age distribution of both the case group and the control group ranged from 16–76 years. In addition, the following groups were excluded: pregnant women, lactating women, those currently suffering from thyroid diseases (e.g., autoimmune thyroiditis), those with direct blood relationships with the case group, and those with a history of mental illness who could not correctly understand the questionnaire (**Figure 1**).

### Estimation of sample size

Multiple studies have shown that the main influencing factors of thyroid nodules include sex, age, radiation, smoking, family history of thyroid disease, and iodine deficiency, among which smoking has a relatively low OR value (15–17). According to the sample size formula, when other variables remain constant, the closer the OR value is to 1, the larger the required sample size. This study selected smoking as a factor for sample size estimation. The OR value of smoking in multifactor analysis and case-control studies of high-frequency ultrasound diagnosis of thyroid nodules was 2.478 (17). Moreover, the sample size estimation was based on an analysis of the prevalence and influencing factors of thyroid nodules in Zhejiang Province, with a smoking exposure rate of p_0_ = 42.09% in the control group (16). The sample size estimation formula was as follows:

$\text{m}={\left[Z_{\alpha }/2+Z_{\beta }\sqrt{P\left(1-P\right)}\right]}^{2}/{\left(P-1/2\right)}^{2}$, where m represents the number of pairs of inconsistent exposure conditions, *P*=*OR*/(1+*OR*), the total number of pairs required M=m/(p_0_q_1_+p_1_q_0_), and p_0_ and p_1_ represent the estimated smoking exposure rates of the control and case groups in the target population, respectively*: p_1_*=*p_0_OR*/[1+*p_0_*(*OR*-1)]

In this study, *α* = 0.05, *Z_α_* = 1.96, *β* = 0.10, *Z_β_* = 1.282, and the values above were put into the formula to obtain the total number of pairs M = 103.

### Thyroid B-ultrasound and questionnaire

A trained physician examined each respondent via a B-type ultrasonic diagnostic apparatus with a probe frequency of 7.5 MHz. The examination was carried out according to the specifications of ultrasonic diagnosis of thyroid nodules (17). A unified questionnaire was used and conducted by trained investigators, who used a one-to-one interview. The main contents of the questionnaire included general information such as age and height; lifestyle behaviors such as smoking and drinking; diseases such as a history of thyroid disorders and diabetes; and radiation, such as X-ray and CT.

### Urine samples, blood collection, and tests of urine iodine and thyroid function

A 5 mL random urine sample and 5 mL peripheral venous blood were collected from each survey subject to detect urinary iodine via the “As-Ce Catalytic Chromatography” method (WS/T 107-2016) (18). A clean polyethylene plastic container with a screw cap was used to collect the urine samples, and the sample volume was less than 5 ml. It could be stored at room temperature for 7 d and at 4°C to 8°C for 1 month. The detection was carried out by professional technicians in the laboratory of the Provincial Center for Disease Control and Prevention. The urine samples and salt samples were stored separately, and they were collected by different people at the same time. The correlation coefficient of the standard curve for urine iodine detection was greater than 0.999. At least one standard substance was inserted for quality control before, during, and after the detection of each batch of samples. When all the measured values of the standard substance were within the control range, the detection results were accepted. Otherwise, the cause was determined, and reinspection was carried out.

The US BECKMAN-access2 analyzer with the thyroid function diagnostic kit was used to detect 8 indicators of serum thyroid function, including serum thyroid stimulating hormone (TSH), free triiodothyronine (FT3), free thyroxine (FT4), triiodothyronine (T3), total thyroxine (T4), thyroglobulin antibody (TgAb), thyroglobulin (Tg), and thyroid peroxidase antibody (TPOAb). Blood was collected aseptically. More than 5 mL of blood (without adding an anticoagulant) was collected. After the blood had completely clotted, it was centrifuged at low speed (2000–2800 rpm for 5–10 minutes), and the serum was transferred to a serum tube (with care to prevent hemolysis). After collection, the samples were stored refrigerated (at 3–5°C). If detection was not completed on the same day, the sample was stored in a low-temperature (-30°C) refrigerator.

### Quality control

This study was approved by the Medical Ethics Committee of the Fujian Provincial Center for Disease Control and Prevention (2017002), and all the respondents provided informed consent. Clean polyethylene plastic containers with screw caps were used for urine sample collection, and at least one reference material was inserted before, during, and after the detection of each batch of samples for quality control. The correlation coefficient of the standard curve of urine iodine detection was above 0.999. Blood was collected aseptically. More than 5 mL of blood was collected (without anticoagulant). After complete coagulation, the mixture was centrifuged at low speed (2000–2800 rpm, 5–10 min), and the serum was aliquoted into 2 mL cryovials.

### Definitions and reference standards

The assessment of iodine nutritional status was performed according to the iodine nutritional status standard recommended by the WHO (19), in which a median urinary iodine concentration <100 μg/L indicates iodine deficiency, 100–199 μg/L indicates adequate iodine, 200–299 μg/L indicates more than adequate iodine, and ≥300 μg/L indicates excessive iodine. The reference ranges of the thyroid function indicators used were as follows (provided by the kit): TSH, 0.340–5.600 µIU/mL; Tg, 1.15–130.77 μg/L; and FT3, 3.80–6.00 pmol/L.

FT4: 7.85–14.40 pmol/L, TT3: 1.34–2.73 nmol/L, TPOAb<9.00 IU/ml, TgAb<4.00 IU/ml, TT4: 78.38–157.40 nmol/L.

### Statistical analysis

SPSS software (SPSS 21.0) was used for data analysis. χ^2^ tested the balance between the case and the control groups in terms of marital status, educational level, and occupation. The Enter method was used to conduct multivariate conditional logistic regression analysis to explore the influencing factors of thyroid nodules further. Moreover, the MUI values of the case group and the control group were tested via the Wilcoxon rank sum test. Urinary iodine and thyroid function indicators that did not conform to the normal distribution were represented by the median and interquartile range. The comparison of thyroid function between the case group and the control group was performed via the Wilcoxon rank sum test, and *P*<0.05 was considered statistically significant.

## Results

### General characteristics of the research subjects

A total of 327 patients with thyroid nodules and 327 controls were included. As shown in **Table 1**, there was no significant difference in the general demographic characteristics between the two groups *(P*>0.05). The two groups were balanced and comparable.

**Table 1:** Comparison of the general demographic characteristics of the case and control groups.

General demographic characteristics	Case group	Control group	*χ*^2^/Z	*P*
Age(y)	54.0(44.0-62.0)	54.0(43.0-63.0)	-0.093	0.926
Marital status			1.013	0.602
Unmarried n (%)	22 (6.7)	23(7.0)		
Married n (%)	283(86.6)	288(88.1)		
Other n (%)	22(6.7)	16(4.9)		
Educational level			1.364	0.505
Elementary school and below n (%)	231(70.6)	223(68.2)		
Middle school n (%)	84(25.7)	86(26.3)		
College and above n (%)	12(3.7)	18(5.5)		
Occupation			0.077	0.782
Nonphysical work n (%)	61(18.7)	60(18.3)		
Manual labor n (%)	266(81.3)	267(81.7)		

### Water iodine concentration and saltwater iodine concentration in the three different regions

Four water samples were collected from each of the areas with adequate iodine intake, areas with mild iodine intake deficiency, and areas with more than adequate iodine intake, and the median water iodine concentrations were 6.5, 6.4, and 2.75 μg/L, respectively. The iodized salt coverage rates in the three regions were 0%, 89.5%, and 100%, respectively; the consumption rates of qualified iodized salt were 0%, 71.60%, and 96.8%, respectively; and the median salt iodine levels were 0 mg/kg, 22.3 mg/kg, and 25.0 mg/kg, respectively (**Table 2**).

**Table 2:** Distribution of the salt iodine concentration in the three regions.

Areas	Sample size	Median salt iodine level (mg/kg)(P25-P75)	Iodized salt coverage rate (%)	Consumption rate of qualified iodized salt (%)	Salt iodine frequency distribution (%)			
					0-	5-	20-	50-
Areas with mild iodine deficiency	95	0	0	0	100	0	0	0
Areas with adequate iodine intake	95	22.3(19.9-24.7)	89.50	71.60	10.5	17.9	71.6	0
Areas with more than adequate iodine intake	95	25.0(23.5-25.9)	100.00	96.80	0	3.2	96.8	0

### Relationship between urinary iodine concentration and thyroid nodules

**Figure 2:**
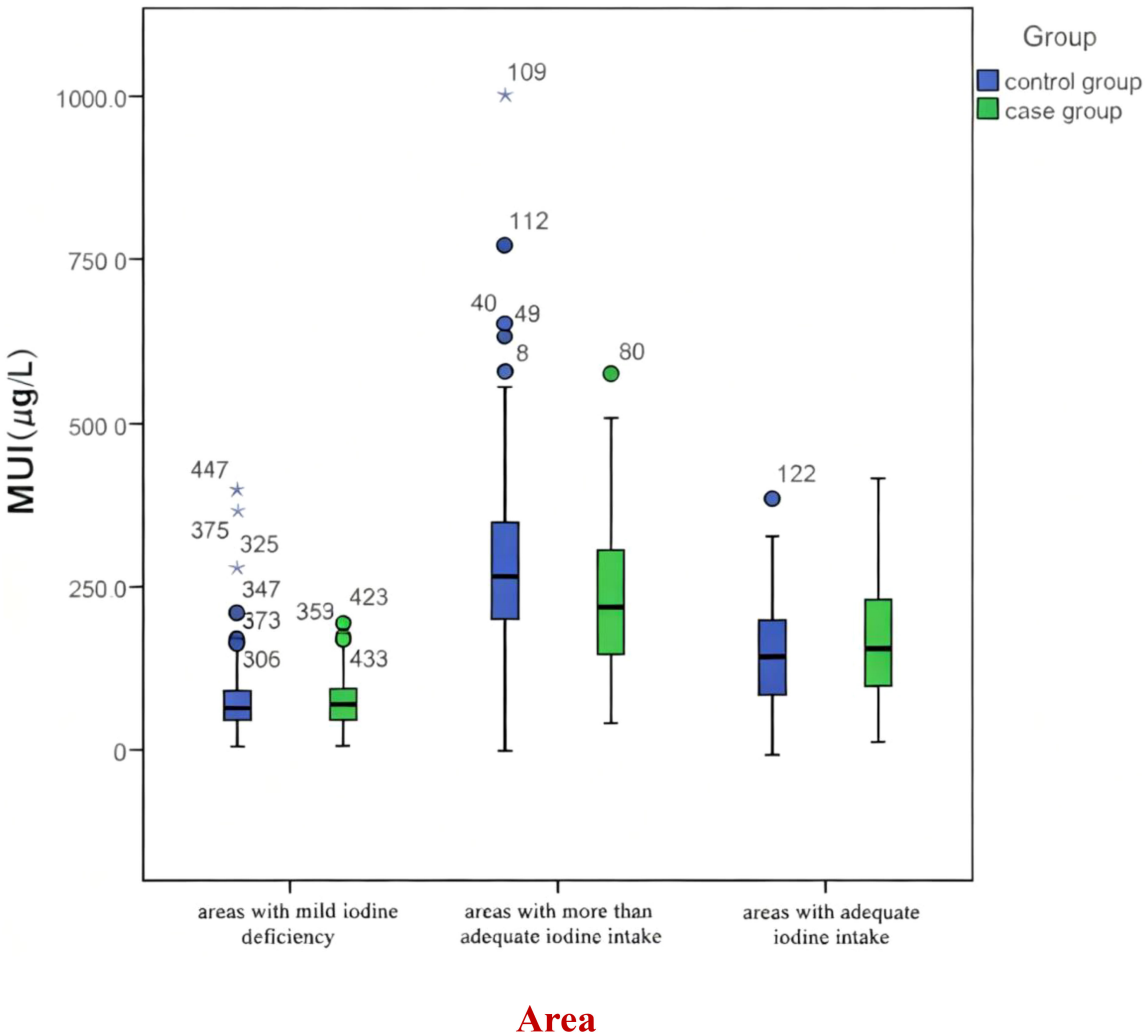
Relationship between urinary iodine concentration and thyroid nodules.

**Figure 3:**
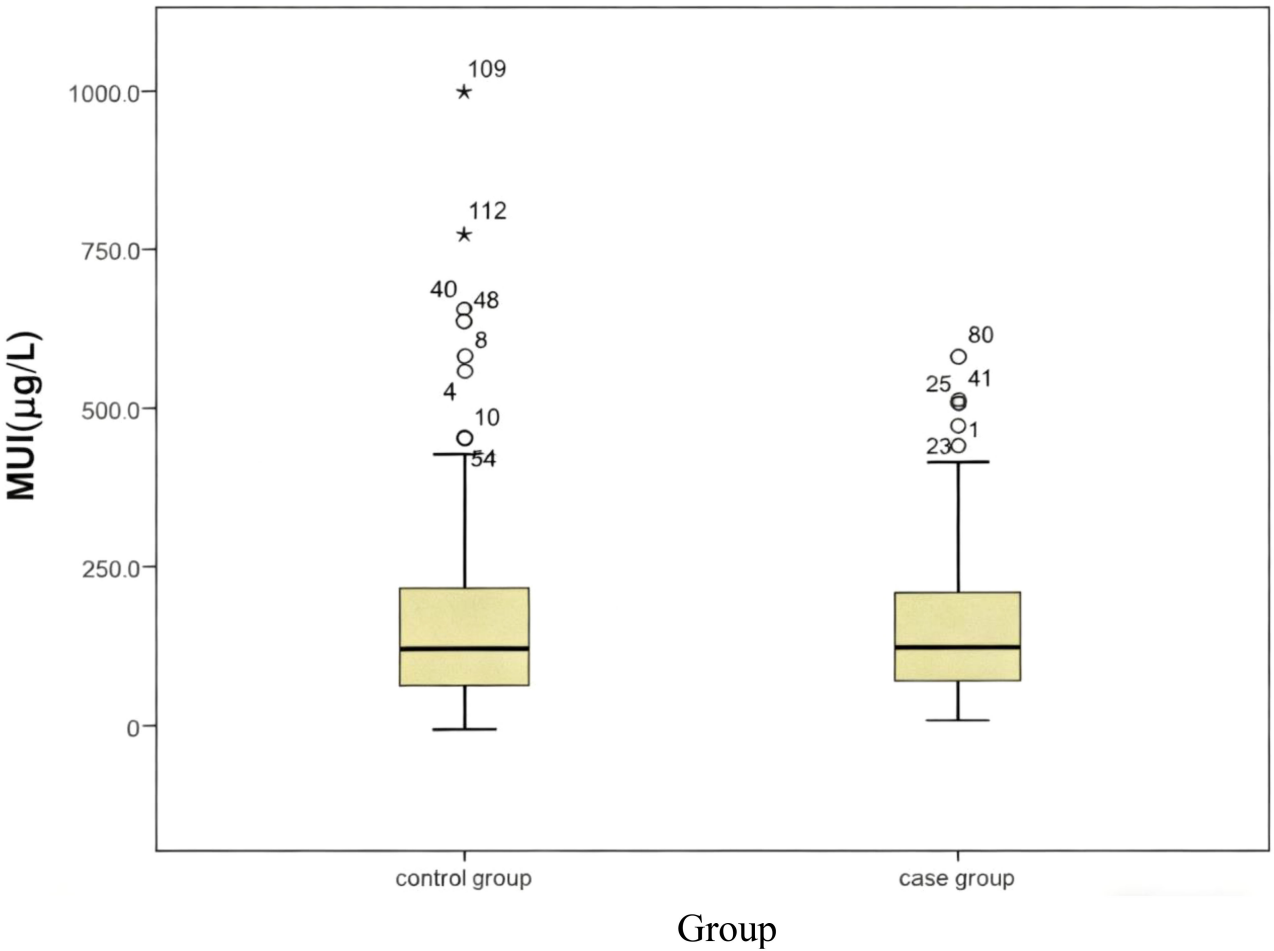
Comparison of urinary iodine concentrations between the case group and the control group.

There was no significant difference in MUI between the case group and the control group in areas with adequate iodine intake, areas with mild iodine deficiency, or the whole population *(P*>0.05). Nevertheless, the MUI of the case group was lower than that of the control group in areas with more than adequate iodine intake (P<0.05). (**Table 3**, **Figures 2**, **3**).

**Table 3:** Relationships between urinary iodine concentration and thyroid nodules.

	Case group MUI (μg/L)(*P25-P75*)	Control group MUI (μg/L)(*P25-P75*)	*Z*	*P*
Areas with adequate iodine intake	154.40 (96.74-229.68)	141.96 (83.40-199.47)	-1.672	0.094
Areas with mild iodine deficiency	70.10 (45.40-93.60)	63.90 (45.80-91.50)	-0.239	0.811
Areas with more than adequate iodine intake	218.10 (142.81-313.36)	265.32 (195.42-352.04)	-1.976	0.048
The whole population	121.18 (69.20-209.35)	119.30 (61.48-216.22)	-0.157	0.875

### Differences in thyroid function indicators between the case group and the control group

The K-S normality test was performed on the thyroid function indicators of the case group and the control group. The data for each indicator did not follow a normal distribution (*P*<0.05). The Tg and FT4 levels of the case group in the areas with mild iodine deficiency, the areas with more than adequate iodine intake, and the whole population were greater than those in the control group (*P*<0.05). Only the Tg level of the case group was higher than that of the control group in the areas with adequate iodine intake (*P*<0.05). Furthermore, other indicators were not significantly different (**Table 4**).

**Table 4:** Comparison of various thyroid function indicators between the case group and the control group.

Thyroid function indicators	Areas with mild iodine deficiency		Areas with adequate iodine intake		Areas with more than adequate iodine intake		The whole population	
	Case group M (P25-P75)	Control group M(P25-P75)	Case group M(P25-P75)	Control group M(P25-P75)	Case group M(P25-P75)	Control group M(P25-P75)	Case group M(P25-P75)	Control group M(P25-P75)
TSH (μIU/mL)	1.51(1.01-2.20)	1.62(1.26-2.19)	1.73(1.21-2.45)	1.79(1.22-2.60)	1.76(1.08-2.94)	1.98(1.47-3.01)	1.64(1.10-2.46)	1.79(1.34-2.57)
Tg (μg/L)	18.20(9.81-30.26)c	13.95(8.66-23.20)	12.97(7.23-24.40)b	9.79(4.82-15.86)	11.69(5.25-23.27)a	6.70(3.96-11.62)	14.10(7.35-25.16)d	9.70(5.16-16.69)
FT3 (pmol/L)	5.21(4.70-5.70)	5.22(4.84-5.60)	5.04(4.70-5.57)	5.24(4.82-5.63)	5.32(5.00-5.76)	5.17(4.84-5.64)	5.22(4.81-5.70)	5.21(4.83-5.62)
FT4 (pmol/L)	12.65(11.50-13.55)c	11.88(11.06-12.85)	12.31(11.16-13.32)	12.04(11.10-13.11)	12.64(11.55-13.66)a	12.11(11.33-13.30)	12.51(11.35-13.54)d	12.03(11.16-13.11)
TPOAb (IU/ml)	1.00(0.50-2.10)	1.10(0.50-2.50)	0.80(0.40-2.20)	1.25(0.50-3.70)	1.80(0.90-4.00)	1.60(0.80-3.27)	1.10(0.50-3.00)	1.20(0.60-3.20)
TT3 (nmol/L)	1.51(1.33-1.78)	1.55(1.39-1.78)	1.43(1.26-1.65)	1.32(1.32-1.68)	1.62(1.43-1.79)	1.57(1.40-1.72)	1.54(1.33-1.76)	1.55(1.37-1.72)
TT4 (nmol/L)	97.75(81.65-109.01)	94.07(81.97-101.10)	92.17(80.65-104.96)	91.27(81.98-99.58)	100.49(86.50-117.75)	97.66(89.62-110.19)	96.20(82.57-111.01)	94.50(84.88-104.02)

a represents the comparison of Tg and FT4 between the case group and the control group in the area with more than adequate iodine intake, *P*<0.05; b represents the comparison of Tg between the case group and the control group in the area with adequate iodine intake, *P*<0.05; c represents the comparison of Tg and FT4 between the case group and the control group in the area with mild iodine deficiency, *P*<0.05; d represents the comparison of Tg and FT4 between the case group and the control group in the whole population, *P*<0.05.

### Relationship between thyroid nodules and thyroglobulin

**Figure 4:**
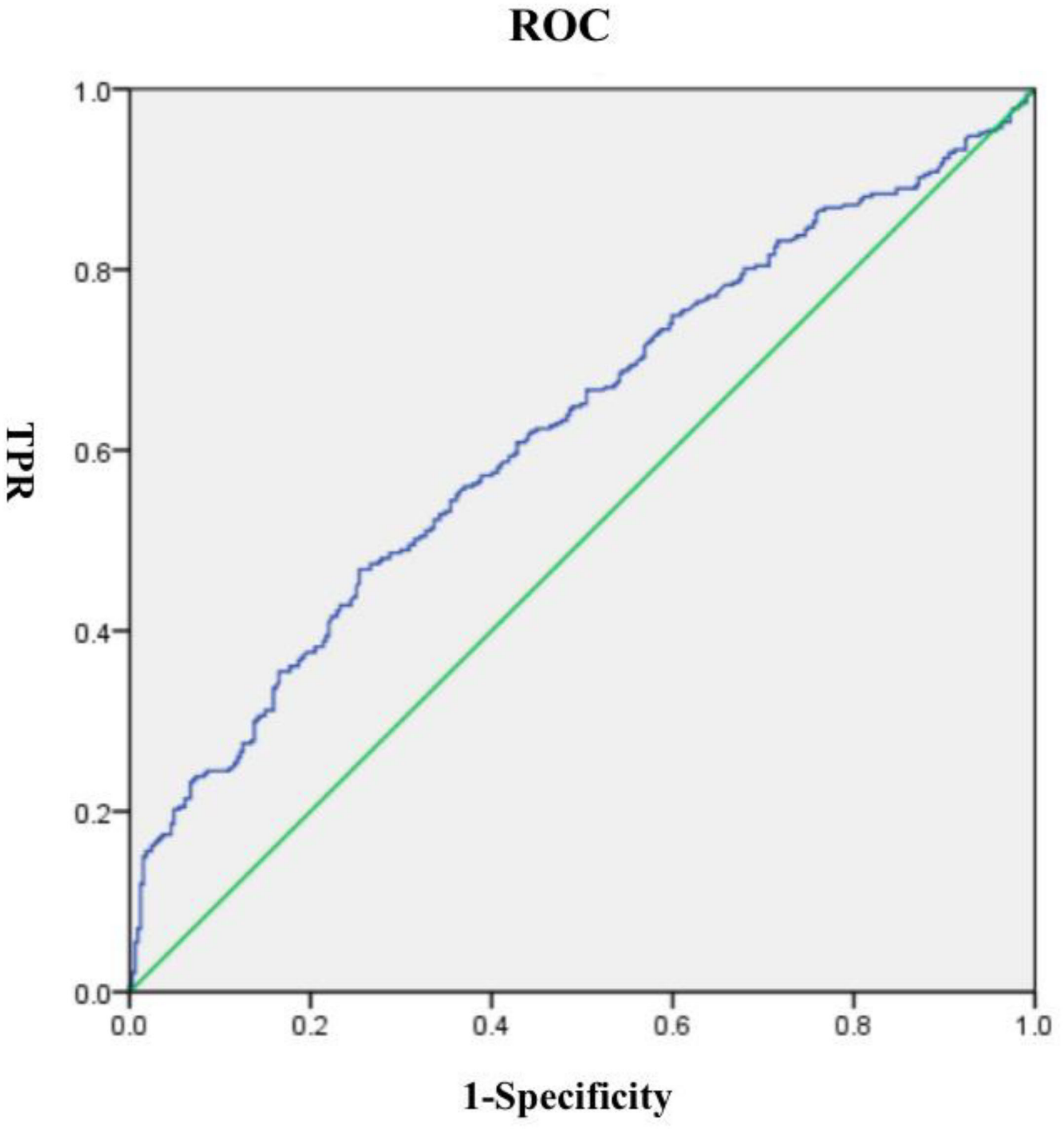
ROC curve analysis for the relationship between thyroid nodules and thyroglobulin (Tg) concentration.

Analysis via the receiver operating characteristic (ROC) curve indicated that thyroglobulin (Tg) is of diagnostic value for distinguishing between the presence and absence of thyroid nodules. AUC = 0.625, P<0.001 (**Figure 4**).

### Relationship between obesity and thyroid nodules

According to univariate conditional logistic regression analysis, there was no significant correlation between obesity and nodule incidence (*P*>0.05). The OR was 1 (**Table 5**).

**Table 5:** Conditional logistic regression univariate analysis of the relationship between obesity and thyroid nodules.

Weight	Regression coefficients	Standard error	Waldχ^2^	*P*	OR(95%CI)
Normal weight group (reference group)					
Underweight group	-0.333	0.335	0.985	0.321	0.717(0.372-1.383)
Overweight overweight	-0.235	0.183	1.649	0.199	0.790(0.552-1.132)
obesity group	-0.339	0.283	1.439	0.230	0.712(0.409-1.240)

### Multifactor analysis

The multivariate conditional logistic regression model was fitted via the Enter method, and the results revealed that a history of thyroid disease, Tg, TgAb, and thyroid nodules were significantly associated (**Table 6**).

**Table 6:** Multivariate conditional logistic regression analysis of the factors influencing thyroid nodules.

Variable	Regression coefficients	Standard error	Waldχ2	*P*	OR(95%CI)
History of thyroid disease					
Unknown	1.686	0.414	16.597	<0.001	5.398(2.398-12.147)
History of thyroid disease	3.009	1.094	7.567	0.006	20.262(2.375-172.872)
Tg					
Below the standard of Tg	-1.353	0.647	4.374	0.036	0.258(0.073-0.918)
TgAb					
Above the standard of TgAb	1.146	0.572	4.010	0.045	3.146(1.025-9.660)

## Discussion

The results of this study revealed that the difference in the MUI between the case group and the control group was statistically significant only in areas with more than adequate iodine intake (*P*<0.05). In both areas with mild iodine deficiency, areas with adequate iodine intake, and areas with more than adequate iodine intake, the Tg level of the case group was higher than that of the control group (*P*<0.05), which may suggest that Tg level detection has certain diagnostic value in distinguishing thyroid nodules. Additionally, the multivariate conditional logistic regression model revealed that a history of thyroid disease, Tg, TgAb, and thyroid nodules were significantly associated.

Studies on the relationship between iodine nutrition and thyroid nodules are currently inconsistent. Some scholars believe that excessive iodine status is a risk factor for thyroid dysfunction and thyroid nodules (20). Kim HJ reported that patients with thyroid cancer were more likely to have relatively low (UIC < 300 μg/L) and extremely high (UIC ≥ 2500 μg/L) iodine nutrition statuses than those with benign thyroid nodules (15). Xiaoming Lou (21) suggested that thyroid nodules have a U-shaped relationship with the urinary iodine concentration. Compared with subjects with relatively low UICs (<100 μg/L), those with UICs between 200–399 μg/L had fewer thyroid nodules, and the risk of thyroid nodules was reduced by approximately 37–57%. Subjects with UICs between 100 and 199 μg/L had a reduced risk of thyroid nodules. In addition, Teng W reported that when the urinary iodine concentration was less than 527 μg/L, thyroid nodules were negatively correlated with the urinary iodine concentration. In contrast, when the urinary iodine concentration is ≥527 μg/L, the urinary iodine concentration is not significantly different from that of thyroid nodules (22). We found that in areas with more than adequate iodine intake, the urine iodine concentration in the nodule group was lower than that in the nonnodule group, which might indicate that in areas with more than adequate iodine intake, a slight increase in the urinary iodine concentration might be associated with a lower risk of thyroid nodules. These results are similar to those of other studies showing that thyroid nodules are related to the iodine nutrition level. Thyroid nodules may cause abnormalities in the synthesis or secretion of thyroid hormones, triggering feedback regulation via the hypothalamic-pituitary-thyroid (HPT) axis. If nodules lead to reduced thyroid hormone secretion, thyroid-stimulating hormone (TSH) levels increase to stimulate thyroid function. Elevated TSH then promotes thyroid gland iodine uptake, directing more iodine in the body toward hormone synthesis and ultimately resulting in a decrease in median urinary iodine (MUI) (23). However, it is not clear when the critical value is reached to affect thyroid nodules. Animal studies and prospective cohort studies can be used to explore further the critical value of the iodine nutrition concentration in affecting thyroid nodules.

Analysis via the receiver operating characteristic (ROC) curve indicated that thyroglobulin (Tg) is of diagnostic value for distinguishing between the presence and absence of thyroid nodules. AUC = 0.625, P<0.001. Fang Gaojie argued that thyroglobulin (Tg) might be involved in the development and progression of thyroid nodules and that Tg level detection has certain diagnostic value in distinguishing between benign and malignant thyroid nodules, which helps identify patients with high-risk thyroid malignant nodules (24). The results of multivariate conditional logistic regression analysis revealed that thyroid nodules were related to Tg, which was consistent with the results of other studies. Dellal FD revealed that Tg was elevated after fine needle aspiration biopsy of thyroid nodules, and the Tg level of malignant thyroid nodules was significantly higher than that of suspected malignant and benign nodules (25). The higher the Tg concentration is, the greater the risk of developing thyroid nodules, indicating that Tg can be used as a functional biomarker for thyroid nodules (26). Elevated Tg levels may impair the function of regulatory T cells, thereby increasing the risk of thyroid nodules and autoimmune thyroid disease (27). The literature has shown that a positive serum TgAb test is an independent predictor of thyroid malignancy in thyroid nodules (10), which is the same conclusion as that of this study.

A history of thyroid disease significantly increased the risk of thyroid nodules, OR = 20.262 (2.375–172.872). A study by Kitahara CM also revealed that the incidence of thyroid disease was positively correlated with the risk of thyroid nodules (malignancy) (28). Our study demonstrated that a history of thyroid disease and thyroid nodules was strongly associated. One of the reasons for the unusually wide confidence interval of the odds ratio (OR) for “history of thyroid disease” might be that the “history of thyroid disease” was not subdivided into specific types (hyperthyroidism, hypothyroidism, nontoxic goiter, etc.). The associations between different thyroid disease types and outcomes might vary significantly; grouping them would lead to “high heterogeneity within the exposed group,” increase the variability of the OR, and thereby increase the confidence interval.

Some current studies have shown that obesity is associated with an increased incidence of nodular thyroid disease (29, 30). However, this study did not draw this conclusion, which might be related to the rural areas with similar living habits selected in this study. The lack of an association between obesity and thyroid nodules in this study might also be related to the fact that the study subjects were from three regions with different iodine nutritional levels. Large differences in iodine nutritional status among the population, without stratified analysis, might offset the impact of obesity. For example, the association between obesity and thyroid nodules may be more evident in iodine-sufficient areas; however, in iodine-deficient or excessive areas, the influence of iodine dominates, leading to the masking of obesity’s effect. Obese individuals are often accompanied by other lifestyle habits (such as a high-sugar diet and a lack of physical activity). Since these factors were not controlled simultaneously in the present study, it might also have been impossible to identify the effect of obesity.

The advantage of this study is that the control group was 1:1 matched with the case group by age (± 3 years), sex, and region, eliminating the influence of these three important confounding factors. The relationships between iodine nutrition and thyroid nodules, as well as other influencing factors, were objectively explored, which is more convincing in terms of causal arguments. Previous studies were mostly limited to cross-sectional surveys or case-control studies with mismatched designs to explore the relationships among thyroid nodules, iodine nutrition, and other influencing factors (20, 31). In addition to common urine samples, blood samples from the survey subjects were also collected, which provided strong evidence for follow-up studies on whether there were differences in the thyroid function of thyroid nodules. At present, relatively few studies have investigated the relationships among Tg, thyroid nodules, and iodine nutrition. This study revealed that Tg level detection has certain diagnostic value in distinguishing thyroid nodules, which is highly important for the clinical diagnosis and treatment of thyroid nodules.

However, there are certain shortcomings in this study. Nodular thyroid diseases do not result from a single factor but rather are the result of interactions among multiple factors, such as heredity and the environment (32). The 1:1 matched case-control study in this study is an analytical epidemiological study, and its causal argument is stronger than that of a cross-sectional study (descriptive epidemiological study) and a mismatched case-control study; however, the chronological sequence of causality cannot be determined; hence, the causality cannot be further verified. Follow-up prospective cohort studies may be conducted to verify the causal relationship between thyroid nodules and iodine nutrition. In this study, the number of case-control pairs was obtained according to the sample size formula, and three survey sites were selected to meet the purpose of the study. However, under practical circumstances, the larger the sample size is, the more convincing the research results. If the sample size and the sampling range can be expanded during research, better research results can be obtained.

## Conclusion

The difference in the MUI between the case group and the control group was statistically significant only in the areas with more than adequate iodine intake. In areas with more than adequate iodine intake, a slight increase in urinary iodine may be associated with a lower risk of thyroid nodules. A history of thyroid diseases and elevated Tg and TgAb levels are risk factors for thyroid nodules. Tg level detection has certain diagnostic value in distinguishing thyroid nodules.

## Data Availability

The original contributions presented in the study are included in the article/supplementary material. Further inquiries can be directed to the corresponding author.
